# A Broad Scope of Differentials: Helicobacter pylori-Associated Chronic Gastritis Mimicking a Gastrointestinal Stromal Tumor

**DOI:** 10.7759/cureus.110230

**Published:** 2026-06-04

**Authors:** Kamal U Azam, Waqas Fazal, Shayan Aziz Khattak, Muhammad Ali, Obaid Ullah

**Affiliations:** 1 Acute Medicine, University Hospital of North Durham, Durham, GBR; 2 Neurology, Royal Stoke University Hospital, Stoke-on-Trent, GBR; 3 Internal Medicine, Salford Royal NHS Foundation Trust, Manchester, GBR; 4 Medicine, Sunderland Royal Hospital, South Tyneside and Sunderland NHS Foundation Trust, Sunderland, GBR

**Keywords:** emergency gastroenterology and endoscopy, gastric hyperplastic polyp, gastrointestinal stromal tumor (gist), h pylori, upper gastro-intestinal bleed

## Abstract

Chronic gastritis is a common manifestation of *Helicobacter pylori* infection. Clinical presentations can range from epigastric discomfort to gastrointestinal bleeding. The clinical course and gross endoscopic findings can mimic alternative diagnoses such as gastric adenocarcinoma and gastrointestinal stromal tumors (GISTs). We report a case of *H. pylori*-associated chronic gastritis that initially presented with frank melena. Subsequent blood tests demonstrated a drop in hemoglobin and a rise in serum urea. The case was managed according to the hospital’s upper gastrointestinal bleed protocols. The next day, endoscopy demonstrated a large polypoidal mass in the gastric antrum suspicious for GIST. However, subsequent biopsy and histological examination showed lymphoid aggregates and active inflammation with comma-shaped bacilli consistent with *H. pylori* infection. The case was subsequently managed with triple eradication therapy. This case underscores the value of prompt management of upper gastrointestinal bleeding and the need to validate diagnoses with definitive modalities to optimize long-term management.

## Introduction

Chronic gastritis is defined as prolonged inflammation of the gastric mucosa and is generally subdivided into atrophic and non-atrophic forms; however, detailed classification can vary. Clinical presentation can vary, ranging from vague abdominal symptoms to more severe cases of gastrointestinal bleeding and chronic weight loss. The global prevalence of chronic gastritis varies considerably, with one study reporting a global incidence rate of gastritis of 27.1 million (95% uncertainty interval = 21.85-33.65) individuals in 2021 [1]; however, this may reflect an underestimate of the true burden. Chronic gastritis is also implicated in the pathogenesis of gastric adenocarcinoma [2,3]. One aspect that was revolutionary in the management of gastritis was the discovery of the microorganism *Helicobacter pylori* [4], a curved Gram-negative bacterium. This enabled the development of targeted therapy to eradicate *H. pylori* and cure chronic gastritis. Early identification and treatment are crucial to avoid progressive complications, as *H. pylori* has been implicated in the development of gastric adenocarcinoma and mucosa-associated lymphoid tissue (MALT) lymphoma. Diagnostic modalities include the urea breath test, stool antigen testing, and upper gastrointestinal endoscopy, biopsy, and direct visualization of the pathogen on histology [5]. In contrast, gastrointestinal stromal tumors (GISTs) are rare pathologies, accounting for 1-2% of gastrointestinal neoplasms. As with gastritis, their presentation can vary from abdominal bloating and pain to gastrointestinal bleeds [6]. These tumors, rather than being caused by bacteria, arise from interstitial cells of Cajal, with the stomach being a common site of origin. Recent studies have identified a possible association between *H. pylori* infection and GIST [7]. However, treatment for GIST is usually a combination of surgery and/or targeted chemotherapy. This overlap in clinical presentation with differences in treatment modality creates a situation of clinical uncertainty. We report a case of *H. pylori*-associated gastritis that mimicked GIST on initial endoscopic assessment.

## Case presentation

A 54-year-old male presented to the emergency department of a district general hospital. He reported a two-week history of persistent black tarry stools and epigastric pains. He also complained of lightheadedness, dyspnea, and palpitations on exertion. He denied any frank hematemesis or hematochezia. On initial assessment, his observations were stable with a blood pressure of 152/87 mmHg, pulse of 82 beats/minute, and oxygen saturation of 98% on room air. Venous blood gas showed a pH of 7.48, paCO_2_ of 3.70 kPa, paO_2_ of 3.0 kPa, potassium of 4.6 mmol/L, lactate of 3.4 mmol/L, and hemoglobin of 82 g/L. A subsequent digital rectal examination showed frank melena but no palpable masses. The remainder of the physical examination did not show any abnormality. On systemic review, he denied any changes to his bowel habits, weight loss, or dysphagia.

On additional inquiry, he had no past medical history and did not use any regular medications or over-the-counter analgesia. He was actively employed as a delivery driver and was a father of three children. He had no smoking history but did drink 10-15 units of alcohol in the form of beer every week, with no reported binge drinking episodes. He reported no family history of malignancy.

Management consisted of intravenous (IV) fluids (lactated Ringer’s), with proton pump inhibitor (PPI) (IV lansoprazole 30 mg BD). He was kept nil by mouth in anticipation of upper gastrointestinal endoscopy. Formal blood tests showed normal renal, bone, and liver function but revealed an elevated urea from 4.4 mmol/L to 6.2 mmol/L, as well as a hemoglobin drop from 152 g/L to 80 g/L. His case was discussed with the on-call gastroenterologist, and he was listed for early morning endoscopy.

**Figure 1:**
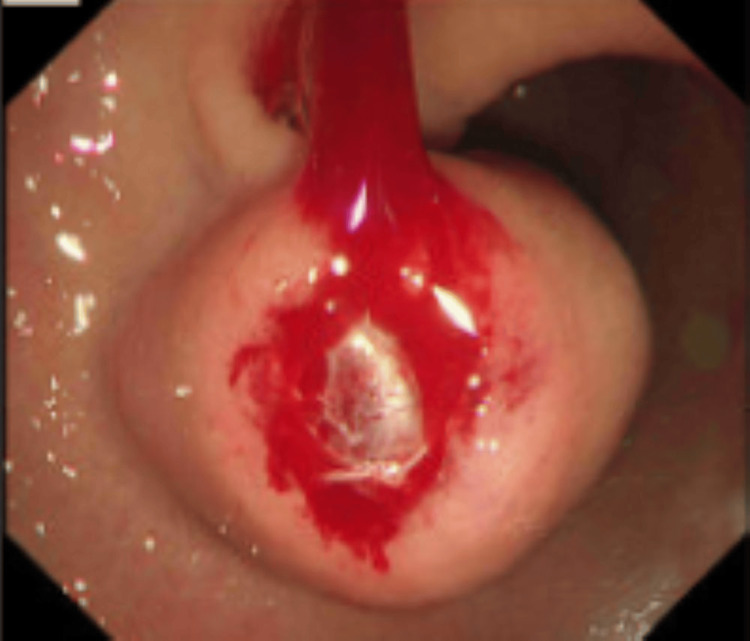
Endoscopic image of an ulcer over the exophytic polyp in the gastric antrum.

Subsequent upper gastrointestinal endoscopy done early morning showed a 2 cm polypoidal lesion between the incisura and antrum of the stomach; there was an ulcer atop the mass with no active bleeding (Figure 1). The appearances were consistent with a GIST. Ten biopsy samples were taken from the lesion. Hemostatic techniques were not used as no active bleeding point was identified. Post-endoscopy, the patient returned to the ward with a plan for IV PPI for three days followed by oral PPI. A CT of the thorax, abdomen, and pelvis with contrast (CT TAP) and a referral to the local upper gastrointestinal multidisciplinary team meeting (UGI MDT) was requested. The patient was allowed to eat and drink. Post-endoscopy blood tests showed a further hemoglobin drop to 66 g/L, and he was transfused with 1 unit of packed red cells, aiming for a target hemoglobin of greater than 70 g/L. Table 1 shows the changes in blood tests over time.

**Table 1:** Variation in blood tests during admission and follow-up.

Blood test	Reference value	Pre-admission	Arrival (day 1)	Pre-endoscopy (day 2)	Post-endoscopy (day 3)	Discharge (day 4)	Follow-up (2 weeks after discharge
Serum sodium	135–145 mmol/L	139	138	142	140	142	139
Serum potassium	3.5–5.4 mmol/L	3.6	4.8	4	4	4.4	4.3
Serum urea	2.5–7.8 mmol/L	4.4	6.2	4.6	4.4	4.3	4.8
Serum creatinine	65.4–119.3 µmol/L	88	86	95	94	94	99
Alanine transferase	7–56 U/L	36	23	16	NA	NA	NA
Total bilirubin	3.4–21 µmol/L	8	7	NA	NA	NA	NA
Serum albumin	35–50 g/L	40	42	39	39	37	34
Hemoglobin	130–180 g/L	152	80	78	66	77	110
White cell count	4–11 × 10^9^/L	7.4	7.4	6.1	6.3	7.8	7
Platelet count	150–450 × 10^9^/L	214	355	233	235	278	320
Hematocrit	41–50%	46	29	29	24	29	36
Prothrombin time	11–13.5 seconds	10.8	10.1	NA	NA	NA	NA
Activated partial thromboplastin time	25–35 seconds	34.9	32	NA	NA	NA	NA

**Figure 2:**
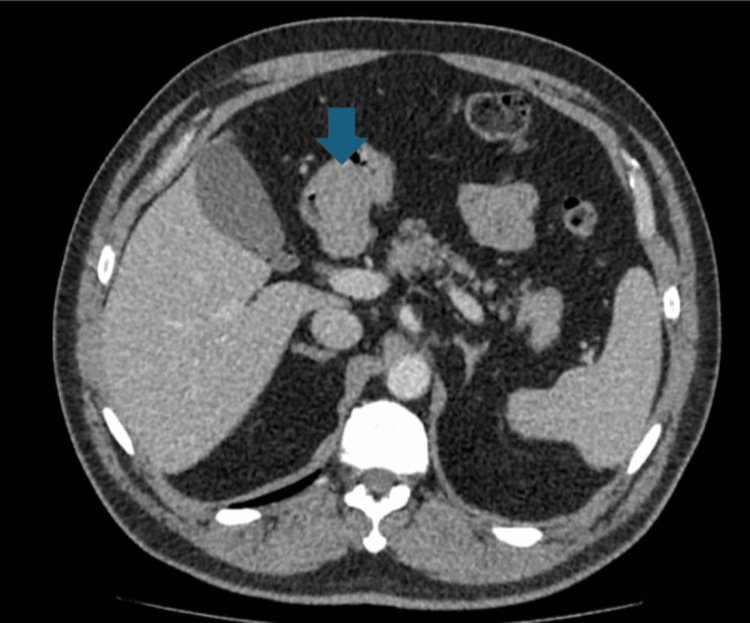
CT of the abdomen (transverse view) with the arrow showing a mass in the gastric antrum.

**Figure 3:**
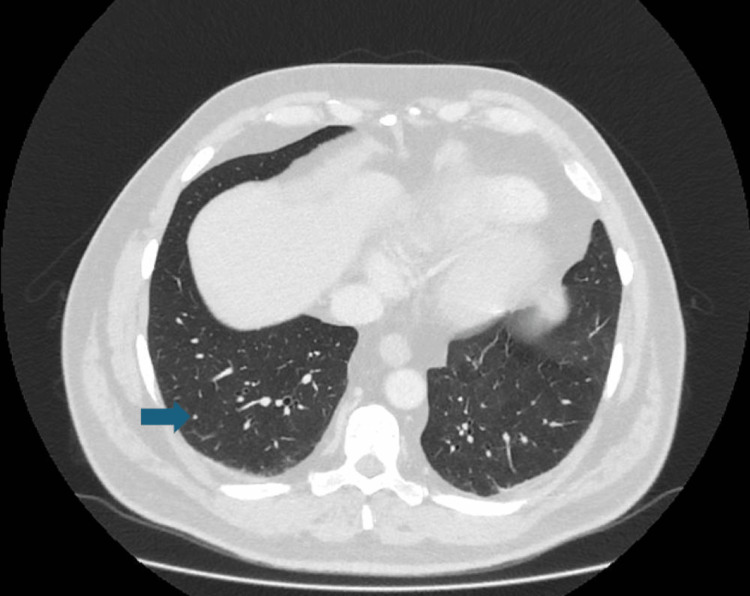
CT of the thorax (transverse view) showing a 3 cm lung mass in the right lower lobe.

CT TAP (Figure 2) showed the same mass in the gastric antrum as well as a 3 cm lung mass in the right lower lobe (Figure 3). With no other abnormalities. Repeat blood tests on day three showed a hemoglobin of 77 g/L.

**Figure 4:**
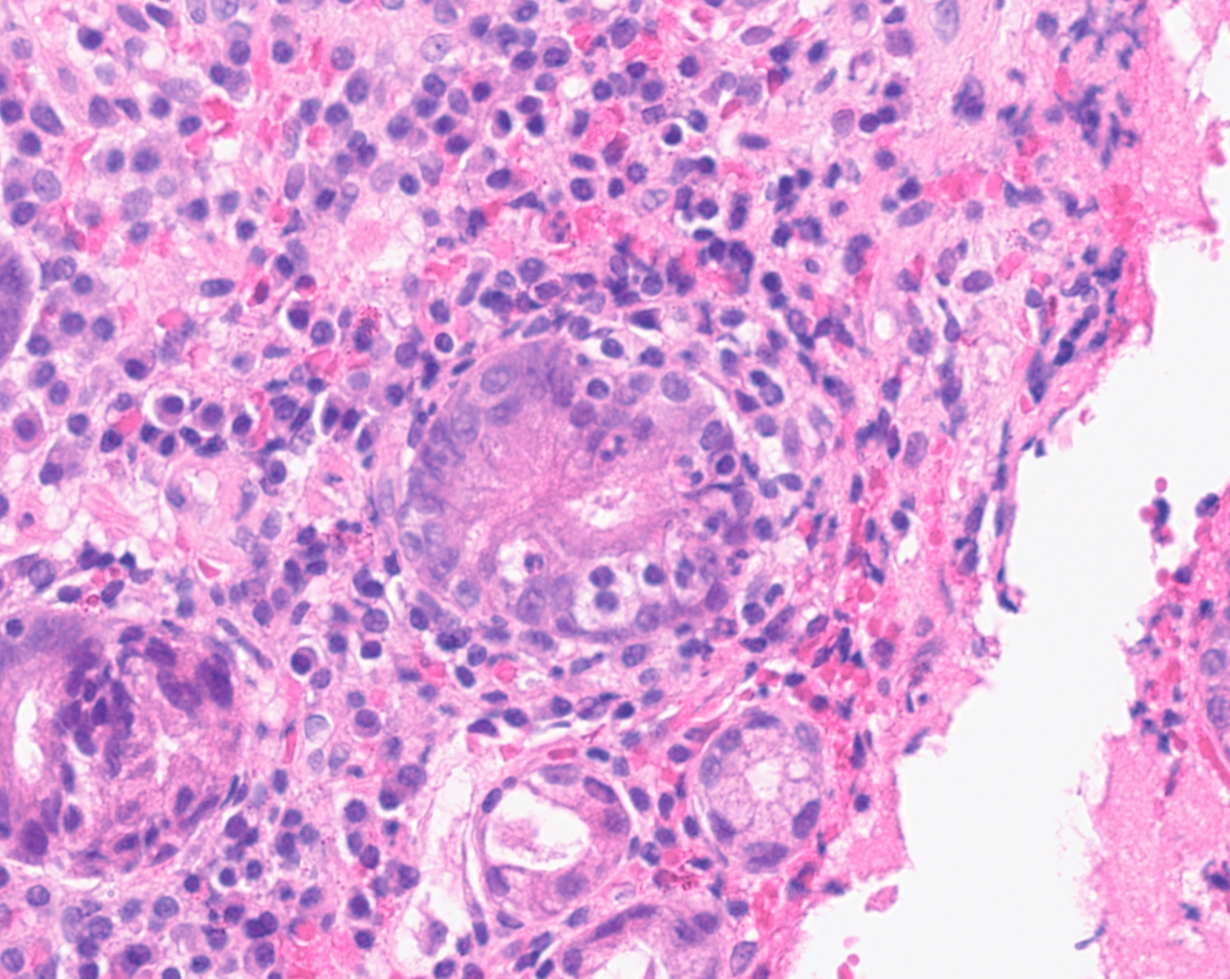
A biopsy slide showing intraepithelial neutrophils and lymphocytes (×43 magnification, hematoxylin and eosin stain).

**Figure 5:**
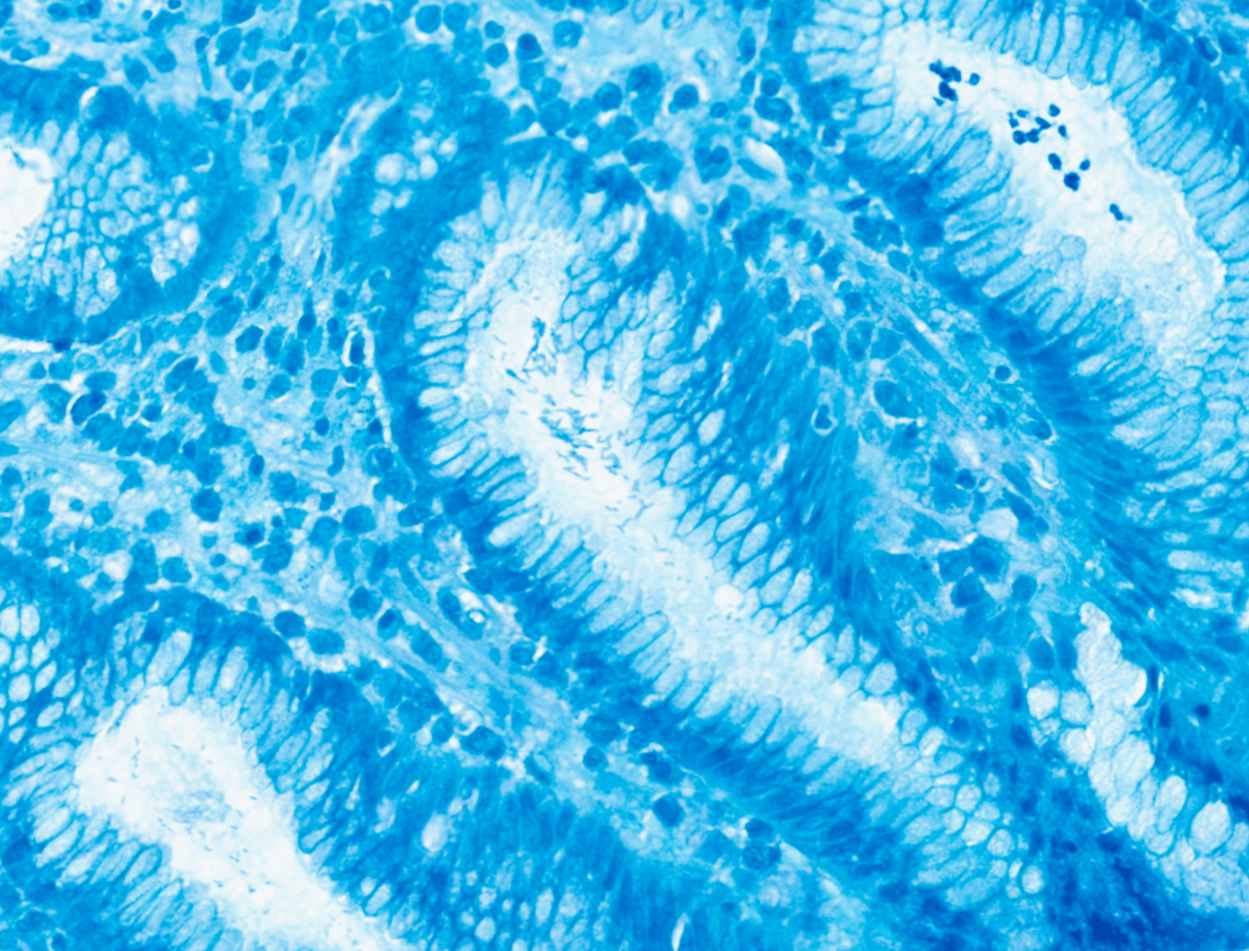
Helicobacter pylori-like organisms seen on biopsy (×35 magnification, modified Giemsa stain).

The UGI MDT outcome was inconclusive, and the case was referred to a tertiary care center for additional support. Histology report from the initial biopsy showed active chronic inflammation, including intraepithelial neutrophil polymorphs and lymphoid cells with no atypical lymphoid infiltrates or reactive lymphoid follicles. The lamina propria contained occasional lymphoid cell infiltrates but no aggregates. In addition, the mucosa fibers were hyperplastic with distorted architecture. A large number of comma-shaped *H. pylori*-like organisms were identified (Figures 4, 5). However, there was no intestinal metaplasia or dysplasia. This was consistent with *H. pylori*-associated active chronic gastritis, with no features to indicate GIST. Therefore, he was discharged on triple therapy for *H. pylori* eradication (oral amoxicillin 1 g BD, clarithromycin 500 mg BD, and lansoprazole 30 mg BD for one week).

**Figure 6:**
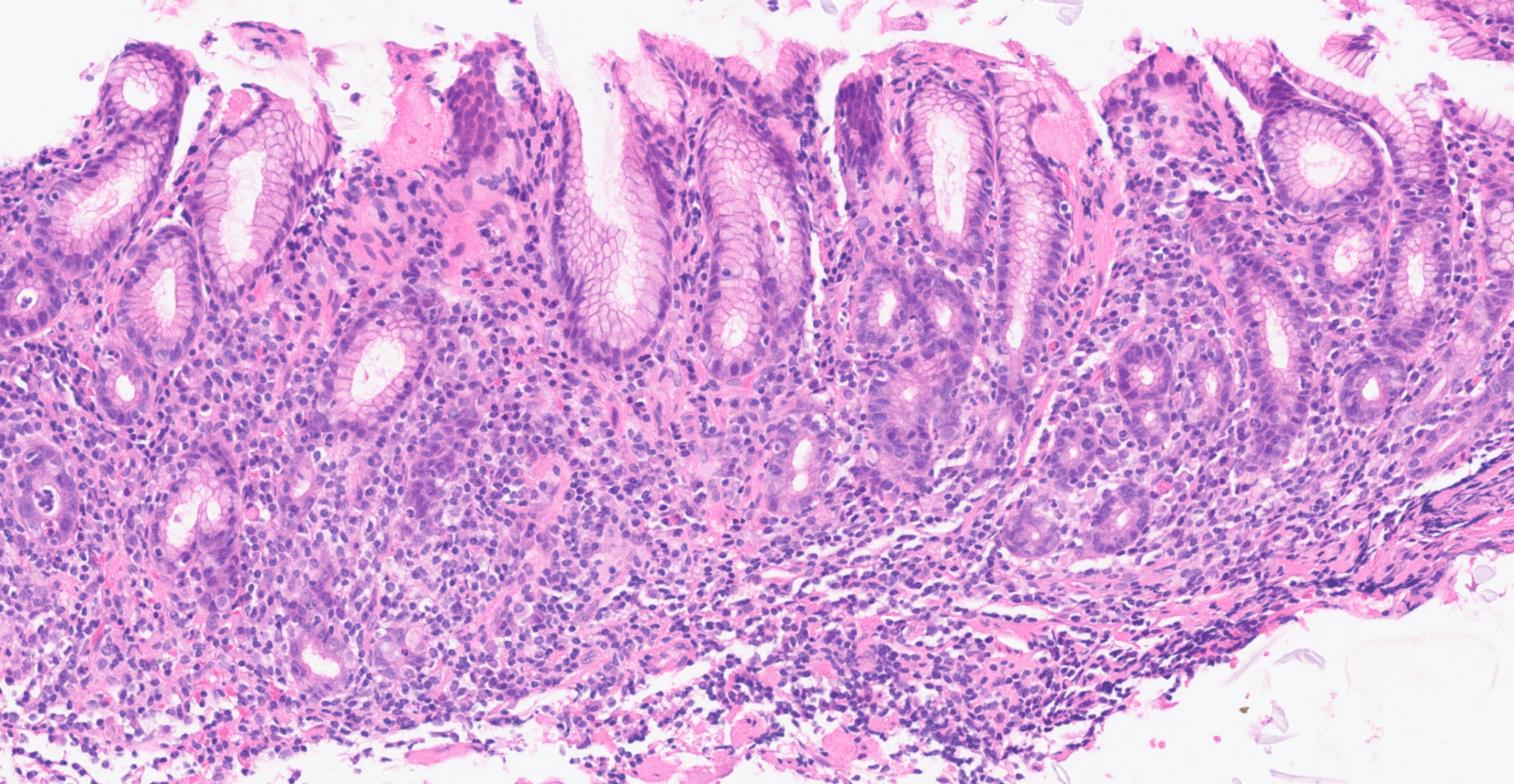
Biopsy showing area of lymphocyte infiltration (×17 magnification, hematoxylin and eosin stain).

Due to the risk of re-bleeding, a repeat endoscopy and polyp removal were scheduled on an outpatient basis in two weeks from discharge. Subsequent follow-up in two weeks revealed a complete resolution of epigastric pain and melena. The subsequent polypectomy was performed without complications. Histology from the fully removed gastric polyp was reported as showing similar intraepithelial lymphoid cells but no persistent organisms or neutrophils, as well as hyperplastic mucosal fibres with distorted architecture. There was no metaplasia or dysplasia (Figure 6). Repeat *H. pylori* testing was not done. Keeping the risk of additional malignant polyps in mind, the choice of colonoscopy was discussed with the patient, for which he consented to a future outpatient colonoscopy in four months from discharge. Colonoscopy revealed no colonic masses or suspicious lesions. A repeat CT TAP after three months revealed a stable lung mass, and he was discharged from any further follow-up.

## Discussion

*H. pylori* infection is a known risk factor in chronic gastritis, and early identification with eradication of the pathogen can change patient outcomes. Common symptoms of chronic gastritis on presentation vary between gastroesophageal reflux, epigastric pain, early postprandial fullness, and gastrointestinal bleeding. Based on clinical suspicion, a test-and-treat strategy is recommended [8], which can be in the form of the rapid urea breath test or fecal antigen. Consideration of an early endoscopic approach is avoided due to minimal patient benefit compared to cost increment [9]. Alternatively, in some instances where disease prevalence is low, empirical treatment can be initiated before testing. Our case represents a situation where disease prevalence was relatively high, but due to the presence of a suspected gastrointestinal bleed, an initial endoscopy approach was appropriate. In the presence of alarm symptoms such as weight loss, dysphagia, overt gastrointestinal bleeding, abdominal mass, or iron deficiency anemia, an early endoscopy is advocated [10]. Further, in situations where *H. pylori* are identified via tissue staining, a dedicated culture is not needed. However, many guidelines [9,11,12] advocate for formal retesting in *H. pylori* infections with high-risk features (gastrointestinal bleeds, masses), as in our case. Resolution of symptoms and normalization of blood tests is not sufficient evidence in these cases. This does underscore a limitation of our case, as retesting on follow-up was not done.

Once the presence of *H. pylori* is confirmed, early treatment is indicated to avoid complications and relieve symptoms. Eradication regimens vary from region to region due to antibiotic resistance patterns, but all regimens advocate for the use of a PPI alongside a dual combination antibiotic regimen of amoxicillin, clarithromycin, and/or metronidazole [11,12]. The value of using bismuth compounds is variable, and its role has mainly been limited to second- or third-line therapy. As in our case, initial management was with lansoprazole and maintenance fluid. However, multiple randomized controlled trials have shown that the use of periprocedural PPI does not equate to improved outcomes [13]; nevertheless, it is still advocated for as per American College of Gastroenterology guidance [14]. Subsequent outpatient management in our case with dual antibiotics and one week of PPI is a proven management strategy for* H. pylori *eradication.

The general pathophysiology of upper gastrointestinal bleeds follows a similar process. Although causes can vary greatly, the loss of blood into the gastrointestinal tract and resultant autodigestion of hemoglobin results in a drop in plasma hemoglobin and elevation of serum urea. Coagulation studies are often needed to look for underlying coagulopathy or factor deficiencies [14,15].

In terms of procedural interventions for upper gastrointestinal bleeding, prompt hemostasis is crucial in predicting patient outcomes. The severity of bleeding and need for urgent inpatient endoscopy can be determined using validated scoring systems such as ABC (Age, Blood tests, Comorbidities), AIMS65 (Albumin, INR, Mental status, Systolic blood pressure Age), Rockall score, and Glasgow-Blatchford Score [15], with higher scores estimating higher mortality and a greater need for inpatient endoscopy. There are many options available to obtain hemostasis, such as adrenaline applicators, thermal ablation, and mechanical clipping, with the most commonly used methods being adrenaline injectors or clipping or a combination of these. Paradoxically, the value of urgent (within six hours) versus early (within 24 hours) endoscopy is unclear. Various studies have shown either no benefit or poorer patient outcomes when performed urgently compared to early [16,17]. In our case, endoscopy was done the next day rather than overnight, but as adequate hemostasis could not be administered due to procedure complexity, a passive wait-and-watch approach was adopted.

The role of *H. pylori* in the pathogenesis of chronic gastritis and, in turn, gastric carcinoma is well established, as is its role in the development of MALT lymphomas and hyperplastic polyps [18]. The proposed mechanism is via alteration in gastric acid secretion physiology resulting in chronic inflammation and virulence factors such as CagA that act as oncoproteins and disrupt cell-to-cell interaction. Indeed, the findings of the mass, as in our case, would warrant the need for biopsy. However, these would not have gross morphological or histological appearances, as in our case. *H. pylori* is implicated in gastric mucosal atrophy or hyperplasia as part of the pathology of chronic gastritis [18] but is rarely associated with the development of benign polyps without hyperplastic features in the gastric mucosa, as seen in our case.

There are few cases where *H. pylori*-associated chronic gastritis presents in gross morphology as an exophytic polypoidal mass, which is more consistent with a GIST or gastric adenocarcinoma. This appearance would warrant the use of cross-sectional imaging such as a CT scan to look for any metastatic features or features of the Carney triad (GIST with pulmonary chondroma and extra-adrenal paragangliomas) [6]; however, the definitive histology findings (the complete absence of epithelioid or spindle cells) and overtly normal CT scan excluded these.

Some studies have shown an association between GIST and *H. pylori* infection [7]; however, complete mimicry is rare. Case reports by Lee et al. [19] and Kathawa et al. [20] reported on unusual presentations of GIST as multiple polyps or exophytic lesions with ulcers, which resembled the gross morphology as seen in our case. The presentation of *H. pylori*-associated chronic gastritis as a large polyp-like mass that resembles GIST is unique.

## Conclusions

Upper gastrointestinal bleeds are a medical emergency that require prompt identification. The value of using proven medical therapies as per guidelines and validated risk stratification scores, followed by early endoscopy, is the cornerstone of management. The causes of upper gastrointestinal bleeds can vary, with gastric ulcers being a common cause. It is important to consider a wide range of differentials when lesions are found on endoscopy and use biopsies to confirm the underlying diagnosis. Appropriate and targeted treatment is important for subsequent management. This case underscores the value of prompt management, definitive investigations, and multidisciplinary follow-up. It also emphasizes the need not to jump to conclusions and judge a book by its cover.

## References

[1] Wang L, Jiang W, Li H (2025). Global, regional, and national burden of gastritis and duodenitis from 1990 to 2021 with projections to 2050: a systematic analysis of the Global Burden of Disease Study 2021. Int J Med Sci.

[2] Schindler R (1966). [Chronic gastritis]. Klin Wochenschr.

[3] Price AB (1991). The Sydney System: histological division. J Gastroenterol Hepatol.

[4] Marshall BJ, Warren JR (1984). Unidentified curved bacilli in the stomach of patients with gastritis and peptic ulceration. Lancet.

[5] Valle J, Seppälä K, Sipponen P, Kosunen T (1991). Disappearance of gastritis after eradication of Helicobacter pylori. A morphometric study. Scand J Gastroenterol.

[6] Menge F, Jakob J, Kasper B, Smakic A, Gaiser T, Hohenberger P (2018). Clinical presentation of gastrointestinal stromal tumors. Visc Med.

[7] Kagihara J, Matsuda B, Young K (2020). Novel association between Helicobacter pylori infection and gastrointestinal stromal tumors (GIST) in a multi-ethnic population. Gastrointest Stromal Tumor.

[8] Howden CW, Hunt RH (1998). Guidelines for the management of Helicobacter pylori infection. Ad Hoc Committee on Practice Parameters of the American College of Gastroenterology. Am J Gastroenterol.

[9] Ford AC, Qume M, Moayyedi P (2005). Helicobacter pylori "test and treat" or endoscopy for managing dyspepsia: an individual patient data meta-analysis. Gastroenterology.

[10] Ikenberry SO, Harrison ME, Lichtenstein D (2007). The role of endoscopy in dyspepsia. Gastrointest Endosc.

[11] Malfertheiner P, Megraud F, O'Morain CA (2017). Management of Helicobacter pylori infection-the Maastricht V/Florence Consensus Report. Gut.

[12] Zagari RM, Romano M, Ojetti V (2015). Guidelines for the management of Helicobacter pylori infection in Italy: the III Working Group Consensus Report 2015. Dig Liver Dis.

[13] Sreedharan A, Martin J, Leontiadis GI, Dorward S, Howden CW, Forman D, Moayyedi P (2010). Proton pump inhibitor treatment initiated prior to endoscopic diagnosis in upper gastrointestinal bleeding. Cochrane Database Syst Rev.

[14] Laine L, Jensen DM (2012). Management of patients with ulcer bleeding. Am J Gastroenterol.

[15] Alali AA, Barkun AN (2023). An update on the management of non-variceal upper gastrointestinal bleeding. Gastroenterol Rep (Oxf).

[16] Lau JY, Yu Y, Tang RS (2020). Timing of endoscopy for acute upper gastrointestinal bleeding. N Engl J Med.

[17] Jafar W, Jafar AJ, Sharma A (2016). Upper gastrointestinal haemorrhage: an update. Frontline Gastroenterol.

[18] Sipponen P, Hyvärinen H (1993). Role of Helicobacter pylori in the pathogenesis of gastritis, peptic ulcer and gastric cancer. Scand J Gastroenterol Suppl.

[19] Lee F, Paik D, Deng Q, Wan D (2025). Bleeding gastric hyperplastic polyps in the setting of lymphocytic gastritis. Am J Gastroenterol.

[20] Kathawa J, Bazzy D, Darany G, Elhaj K (2025). An atypical presentation of a newly found GIST tumor. Am J Gastroenterol.

